# Designing and Fabrication of Nano-Hydroxyapatite and Curcumin-Loaded Chitosan/PVA Nanofibrous Mats for Potential Use as Wound Dressing Biomaterials

**DOI:** 10.3390/nano15020082

**Published:** 2025-01-07

**Authors:** Amira M. EL-Rafei, Giorgia Maurizii, Annalisa Aluigi, Giovanna Sotgiu, Marianna Barbalinardo, Tamara Posati

**Affiliations:** 1 Refractories, Ceramics and Building Materials Department, National Research Centre, 33 EL Bohouth St., Dokki, Giza P.O. Box 12622, Egypt; 2 Department of Biomolecular Sciences, University of Urbino Carlo Bo, Piazza del Rinascimento, 6, 61029 Urbino, Italy; g.maurizii@campus.uniurb.it (G.M.); annalisa.aluigi@uniurb.it (A.A.); 3 Consiglio Nazionale delle Ricerche (CNR), Istituto per la Sintesi Organica e Fotoreattività (ISOF), Via Piero Gobetti, 101, 40129 Bologna, Italy; giovanna.sotgiu@isof.cnr.it (G.S.); marianna.barbalinardo@ismn.cnr.it (M.B.); tamara.posati@isof.cnr.it (T.P.)

**Keywords:** chitosan, polyvinyl alcohol, nano-hydroxyapatite, curcumin, electrospinning, drug delivery

## Abstract

Chitosan/polyvinyl alcohol nanofibrous mats loaded with nano-hydroxyapatite and/or curcumin are successfully fabricated by the electrospinning method for the first time. Nano-hydroxyapatite is prepared by the co-precipitation method. The XRD pattern of calcined powder at 700 °C for 2 h reveals the presence of hydroxyapatite as a sole phase. FT-IR confirms its purity. The morphology of the hydroxyapatite is studied by HR-TEM. Nano-hydroxyapatite and curcumin are added at 5 wt% with respect to the polymer weight. XRD, FE-SEM, FT-IR, and HR-TEM are used to characterize the fabricated nanofibrous mats. The results confirm the successful loading of nano-hydroxyapatite and curcumin within the fabricated mats. The in vitro antimicrobial results show that most of mats have significant antimicrobial effects against *E. coli* and *S. aureus*. The fabricated matd are biocompatible with fibroblasts and the presence of curcumin increases cell viability. Curcumin release from both CS/PVA/Cur and CS/PVA/HA/Cur nanofiber mats principally follows the Korsmeyer–Peppas and Peppas–Salhin models.

## 1. Introduction

Innovative treatments for open wounds aim to control bleeding and inflammation. In the 1960s, wound dressings played a minimal role in the enhancement of the wound-healing process. Winter succeeded in developing the first bioactive wound dressing in 1962, which is composed of biopolymers that provide ideal environments for wound healing. Following that, a revolution in the treatment of skin wounds occurred using these biologically functional materials.

These materials can manage wound exudates, hence, reducing the amount of necrosis on the wound surface, promoting cell development, and acting as a physical barrier against environmental fluctuations. In addition to its mechanical durability and antimicrobial properties, it helps fight common infections and encourage tissue regeneration [1].

Curcumin, Cur, an active ingredient of turmeric, is a naturally occurring poly-phenolic compound and a non-toxic compound [2,3,4,5]. It shows a range of significant biological functions, including being an antibacterial against most pathogenic and nonpathogenic bacteria [2], anti-cancer [3], antifungal [4], anti-oxidant, and anti-inflammatory agents [2,3,4,5,6]. Moreover, it helps modulate collagen in the wounded area and thus enhances wound healing in addition to nerve healing [2,6]. Additionally, curcumin shows chromogenic phenomena, making it a promising chemical sensing compound [4]. However, curcumin is a hydrophobic compound and hence has poor aqueous solubility, which decreases its bioavailability. So, a carrier for the curcumin drug is needed to obtain a realistic therapeutic system and at the same time improve the drug release profile from this carrier to avoid the drug bursting. Nanofibers are considered a good carrier for curcumin for that purpose.

The treatment of wound infections with drug-loaded nanofibers can increase the concentration of the drug at local sites while at the same time decreasing the drug dosage, thereby decreasing its adverse side effects [3]. Nanofibers are associated with ideal physical properties, including a large surface-to-volume ratio, a high aspect ratio, a three-dimensional (3D) architecture, extremely high porosity, and excellent biomimetics of the natural extracellular matrix (ECM), which promote cell adhesion and migration, differentiation, and proliferation; they also can incorporate a drug within the nanofiber mats to improve the healing process of the wound [7,8,9,10]. Emerging biomedical uses for these nanofibers include wound dressing, engineered tissue, biosensors, and controlled drug delivery systems [7,8,9,10].

Such a fine structure with large porosity is an advantage that facilitates the exchange of fluids and gases between the wound and the surrounding environment and at the same time does not allow the passage of bacteria due to its small size compared to bacteria [11]. In addition, studies have shown that some polymeric nanofibers adhered and were biocompatible with hydrated wounds. Thus, the large surface area is suitable for retaining liquids and, thus, the medicine that can be delivered to these wounds.

Electrospinning has quickly grown as an attractive technique to generate polymeric nanofibrous mats. Moreover, the electrospinning process is a simple, low-cost, and green technique. Furthermore, this technique has been successfully used with a wide range of either natural or synthetic polymers [8,9,10]. The morphology and properties of nanofibers depend on three main parameters; first, the polymeric solution parameters, i.e., concentration, viscosity, surface tension, and conductivity; second, the equipment parameters, i.e., applied electric voltage, distance between needle tip and collector, and shape of the collector; third, the ambient parameters, i.e., temperature, humidity, and atmospheric pressure [12,13]. In this technique, viscoelastic polymer solutions are elongated using an electric field and solidified on the collector to form nanofiber membranes [10,13].

The scientific community is becoming very interested in the usage of nanofillers rather than microfillers inside a polymer matrix, thus achieving more controllable drug release kinetics. Moreover, improvement in mechanical properties and expansion into new application areas offer novel biological, electrical, optical, and magnetic functionalities by including determinate fillers [1].

Hydroxyapatite (HA) is identical to the inorganic composition of natural bone tissues; it has been used in tissue engineering to replace missing bone tissue [10,11,12,13,14]. Furthermore, hydroxyapatite has been employed as an appropriate medium for osteoblast seeding and nutrient diffusion for the well-being of the cells.

Moreover, nano-hydroxyapatite (HA) can be used as a nanofiller for the nanofibers, so it can enhance mechanical properties, promote faster bone regeneration, and induce stem cells to differentiate into osteocytes [15]. In spite of its low affinity toward drug molecules, which may result in the burst release of the drug, HA can be used as a drug carrier when loaded in a nanofiber matrix, as the polymer works as a physical barrier, preventing the burst release of the drug [1].

The polymers used in this proposed study have FDA-approved biocompatible materials, including chitosan (CS) and polyvinyl alcohol (PVA) [16]. CS is a naturally occurring polymer of polysaccharides that is nontoxic, biocompatible, biodegradable, and renewable. Moreover, CS is characterized by unique anti-inflammatory and antibacterial properties [17,18]. Unfortunately, due to the limited solubility of CS, and its high solution viscosity, it is frequently combined with other polymers, such as polyvinyl alcohol (PVA), which is a synthetic, non-toxic, water-soluble, biocompatible polymer [14,19,20,21,22].

Several studies have been reported on curcumin [2,3,5,23,24,25,26,27,28,29,30,31,32,33] and chitosan/PVA’s [25,34,35,36] medicinal effects; however, only a few studies have been reported on the wound-healing effects of curcumin-loaded CS/PVA and hydroxyapatite-loaded CS/PVA nanofibers individually, and at the same time, their combination has not been studied. Here, we fabricate CS/PVA nanofibers loaded with curcumin and hydroxyapatite by the electrospinning technique to assess their medicinal characteristics. Delivery of curcumin was achieved by using chitosan/PVA nanofibers with various molecular weights of chitosan. Curcumin’s release time was extended to 148 h [32]. At a neutral pH, the curcumin release pattern from the chitosan–polyvinyl alcohol–genipin–curcumin films showed an initial burst release. The release gradually decreased and exhibited a sustained release after 10 h. This behavior was unaffected by pH variations [31]. The addition of curcumin enhances the antimicrobial activity against *E. coli* when curcumin is added to chitosan/PVA silver nanocomposite films, as compared to curcumin and chitosan/PVA silver film alone [33].

Hydroxyapatite–gelatin/curcumin nanocomposites exhibited notable antimicrobial activity against all tested bacteria. The effectiveness of curcumin against the selected bacterial strains indicates that herbal antimicrobials could potentially replace chemical antibiotics in the future, helping to mitigate bacterial resistance [37].

PLA was effectively reinforced with 1%, 5%, and 10% HAp and plasticized using curcumin extract. The immobilization of curcumin extract enhanced both the mechanical strength and antioxidative properties of the composite [38].

In the proposed study, curcumin (Cur) is used as a model antimicrobial (drug) and HA is used as a drug carrier to evaluate the potential application of the Cur and/or nanohydroxyapatite-loaded chitosan/PVA nanofibers for promising wound dressing applications with enhanced antimicrobial activity.

## 2. Materials and Methods

### 2.1. Materials

Chitosan, CS, (Mw 190,000–310,000 Da, 82.6% degree of deacetylation, ref 448877, Sigma-Aldrich, St. Louis, MO, USA), polyvinyl alcohol, PVA, (Qualikems, Vadodara, India, Degree of polymerization 1700–1800, with hydrolysis degree of 98.99%), acetic acid, Ca (NO_3_)_2_·4H_2_O, and (NH_4_)_2_HPO_4_ (Sigma Aldrich).

Fetal bovine serum (FBS), MEM Non-Essential Amino Acids (NEAA), Dulbecco’s phosphate-buffered saline (DPBS), Resazurin sodium salt, and Dulbecco’s modified Eagle medium (DMEM) were purchased from Merk (Darmstadt, Germany).

### 2.2. Preparation of Nano-Hydroxyapatite

Nano-hydroxyapatite was prepared using published methods via a wet chemical precipitation procedure, from (NH_4_)_2_HPO_4_ and Ca(NO_3_)_2_·4H_2_O solutions, based on the stoichiometric of hydroxyapatite (Ca_10_(PO_4_)_6_(OH)_2_) using the published method [39].

The final dried precipitated of HA was calcined at 700 °C for 3 h in ambient air with a heating rate of 10 °C min^−1^. Then, the calcined powder was ground with agate mortar to be added to the CS/PVA blend.

### 2.3. Preparation of Nanofiber Mat Blends

**Figure 1:**
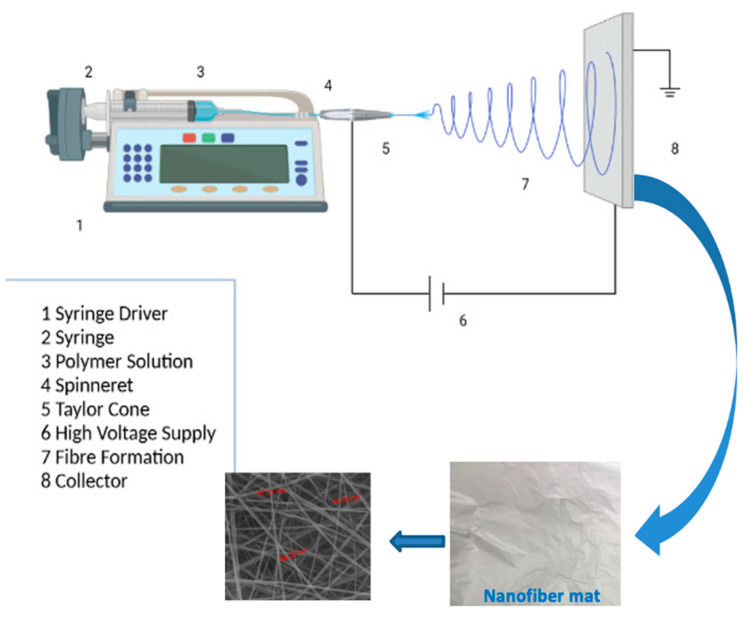
Flow chart of the procedure for fabrication of different compositions of electrospun mats by electrospinning.

The solutions of CS and PVA were prepared as previously reported [22]; in brief: 0.23 g of chitosan was dissolved in 50 mL of 1% aqueous acetic acid and heated at 70 °C with vigorous stirring for 2 h until complete dissolution, then 3.5 g of PVA was added to this solution with vigorous stirring to obtain a homogeneous CS/PVA spinning solution. This base solution to which a 5 wt% of HA, 5 wt% of Cur, and 5 wt% of both of them, based on the solid content of the polymer in base solution, were (loaded) mixed and magnetically stirred for 2 h at room temperature to obtain electrospinning solutions for preparing CS/PVA/HA, CS/PVA/Cur, and CS/PVA/HA/Cur nanofiber mats. The formed solution was transferred into a syringe equipped with a metallic capillary nozzle connected to a high-power supply. The voltage was adjusted to 30 kV. The inner diameter of the used nozzle was 0.49 mm and its height from the aluminum flat static collector was set at 10 cm. The selected flow rate was 0.5 mL/h and the ambient conditions were a temperature of 25 °C and a humidity level of 54%. A flowchart of the proposed methodology is shown in Figure 1.

### 2.4. Characterization

The X-ray powder diffraction (XRD) measurements of samples were carried out on a Rigaku D/Max-3c X-ray diffractometer with CuKα radiation and operated at 40 kV, 50 mA. FT-IR spectra of synthesized samples were also recorded by FT-IR–ATR Brucker Vertex 80 V (Billerica, MA, USA) with a resolution of 4 cm^−1^ in the range of 4000–500 cm^−1^.

The FE-SEM (QUANTA FEG, 250, Capelle Aan Den Ijssel, The Netherlands) equipped with energy-dispersive X-ray spectroscopy (EDS) was used to study the morphology and particle sizes of synthesized samples. Fiber diameters were measured using Image J 1.40G software. Different FE-SEM micrographs for each sample were randomly selected and analyzed.

Transmission electron microscope (TEM) JEM-2100 (JEOL, Tokyo, Japan) was used to examine the nano-hydroxyapatite and the nanofibers. To obtain test samples during the electrospinning process before HR-TEM observation, the copper mesh was placed on the aluminum foil for about 5 s.

### 2.5. Antimicrobial Activity

The antimicrobial activity of Fabricated nanofibers of 5 mm × 5 mm square samples was evaluated against Gram-negative bacteria *Escherichia coli* (ATCC 25922), Gram-positive bacteria (*Staphyllococus aureus* (ATCC 6538)), and pathogenic yeast *Candida albicans* (ATCC 10231) using the disc diffusion method.

These qualitative evaluations were carried out in nutrient agar plates according to [40,41,42]. The nutrient agar medium [fluka, code: 70148)] is a general culture medium that is used for cultivation of less fastidious microorganisms as well as for permanent cultures; it is a dehydrated powder that is composed of (g/L) yeast extract, 2.0; Peptone, 5.0; Meat extract, 1.0; NaCl, 5.0; and Agar, 15.0; for using this medium, 28.0 g was dissolved in one liter of distilled water, then the medium solution was sterilized using an autoclave at 121 °C for 15 min. All microorganisms were inoculated using fresh overnight nutrient broth cultures that were incubated at 37 °C [43,44,45].

Both the bacterial and yeast suspensions were inoculated into each plate containing 20.0 mL of sterile nutrient agar medium (NA) using 25.0 µL from the previously nutrient broth cultures of pathogenic strains, which was adjusted to 0.5 McFarland standard (1.5 × 10^8^/mL). The prepared tested samples were placed on the surface of the previously prepared inoculated agar plates. These inoculated plates were placed in the refrigerator for one hour to allow the active ingredients present in these tested samples to be released and diffused more widely, followed by an incubation of them at 37 °C for 24 h, and zones of inhibition (ZI) were measured in mm [42].

### 2.6. In Vitro Drug Release Studies

The release profile of the electrospun CS/PVA/Cur and CS/PVA/HA/Cur nanofiber mats was evaluated in a Phosphate-Buffered Saline (PBS pH = 7.4) solution with the addition of 0.5% *w*/*v* Tween^®^ 80. The samples were weighed and immersed in sealed glass vials containing 5 mL of release medium. The vials were kept at 37 °C and under gentle stirring for 48 h. At specified time points (0.5, 1, 2, 4, 6, 24, and 48 h), 0.5 mL of release medium was withdrawn and an equal volume of fresh one was added. The amount of Curcumin released at different time points was determined with high-performance liquid chromatography (HPLC, Agilent 1260 Infinity II, Agilent, Santa Clara, CA, USA). The release profile of each sample was studied in triplicate.

#### 2.6.1. HPLC Method for the Quantitative Analysis of Curcumin

The amount of Curcumin released from the electrospun CS/PVA/Cur and CS/PVA/HA/Cur nanofiber mats was evaluated by HPLC. For HPLC analysis, a solution of 0.1% phosphoric acid in water and acetonitrile were used as mobile phases (ratio 55:45), with a flow rate of 0.8 mL/min in an Agilent ZORBAX Eclipse XDB-C18, 250 mm × 4.6 mm, 5 µm column (Agilent, USA). The injection volume was 20 µL, and the detection was recorded at a wavelength of 425 nm, keeping the analysis system at room temperature. A calibration curve of Curcumin was performed using solutions at concentrations in the range of 0.001 to 0.1 mg/mL with a correlation coefficient (R^2^) of 0.9965.

#### 2.6.2. Mathematical Modeling of the Kinetics Release

The release kinetics of curcumin from the electrospun CS/PVA/Cur and CS/PVA/HA/Cur nanofiber mats were studied by using the first-order, Higuchi, Korsmeyer–Peppas, and Peppas–Salhin mathematical models. The fitting of the release profiles was performed with the Origin Software (Origin Pro 2021) and the best-fitting model was chosen considering the adjusted-R^2^ (adj-R^2^).

### 2.7. Cell Cultures and Cells Viability

Mouse embryonic fibroblast cells, NIH-3T3, were cultured under standard conditions standard as per protocol [46]. The cells were seeded in 24-well plates at a concentration of 10 × 104 cells ml and an incubation time of 24 h.

Cells were seeded on CS/PVA, CS/PVA/HA, CS/PVA/Cur, and CS/PVA/HA/Cur with complete medium. Cell viability was determined by resazurin reduction assay, the protocol followed is described by Babalinardo et al. [47].

## 3. Results and Discussion

### 3.1. Characterization of Nano-Hydroxyapatite

#### 3.1.1. XRD Analysis

**Figure 2:**
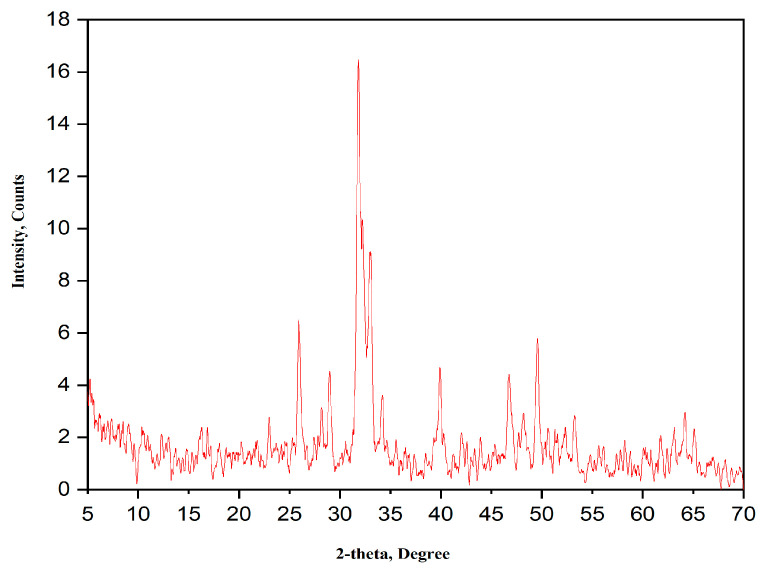
XRD pattern of the as-prepared HA powder calcined at 700 °C for 3 h.

The XRD pattern of the calcined HA is shown in Figure 2. The XRD pattern shows all the HA characteristic peaks according to PDF # 09-432; however, the corresponding X-ray peaks are broadened due to the smaller size of the crystallites. According to Scherrer’s equation, the size of HA crystallites obtained from XRD analysis is ~21 nm.

#### 3.1.2. HR-TEM

**Figure 3:**
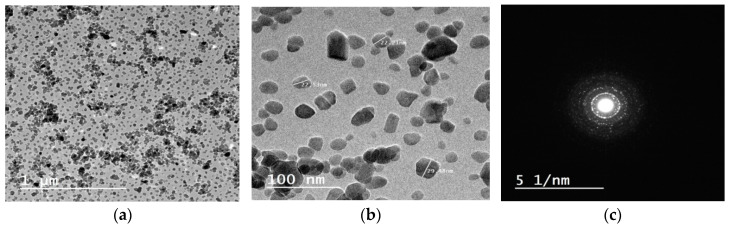
HR-TEM micrographs of HA nanoparticles at (**a**) low magnification, (**b**) high magnification, (**c**) the corresponding selected area electron diffraction (SAED).

The HR-TEM micrograph of synthesized HA powder is shown in Figure 3. As can be seen, the nanoparticles have a semi-spherical morphology with crystal sizes ranging from 10 to 50 nm, (Figure 3a,b).

The selected area electron diffraction (SAED) indicates the crystalline nature of the prepared HA, (Figure 3c).

#### 3.1.3. Fourier Transform Infrared Spectroscopy

**Figure 4:**
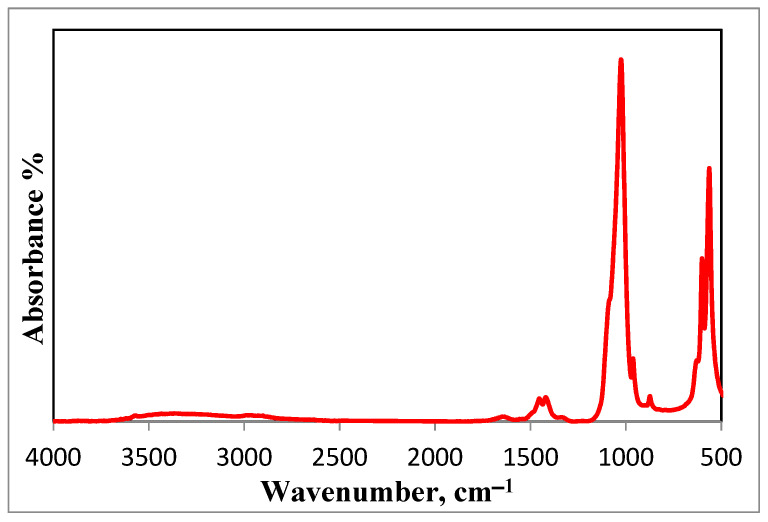
FT-IR spectrum of the prepared HA nanoparticles.

The FT-IR spectrum of HA is shown in Figure 4. The FT-IR spectrum confirms that the preparation of HA is in its pure form, as all the absorption bands are assigned to HA, namely; 563, 600, 874, and 1025 cm^−1^ bands. Bands at 1647 and 3366 cm^−1^ are assigned to the presence of water. Bands at 1454 and 1420 cm^−1^ are attributed to the stretching vibrations of the phosphate group. Bands at 1025 and 964 cm^−1^ exist because of the stretching mode of a phosphate group. Moreover, the band at 874 is attributed to the carbonate group. Whereas bands at 600 and 563 cm^−1^ are due to bending vibrations of the phosphate group [48,49].

### 3.2. Characterization of As-Spun Mats

#### 3.2.1. Nanofiber Morphology and EDS Analysis

**Figure 5:**
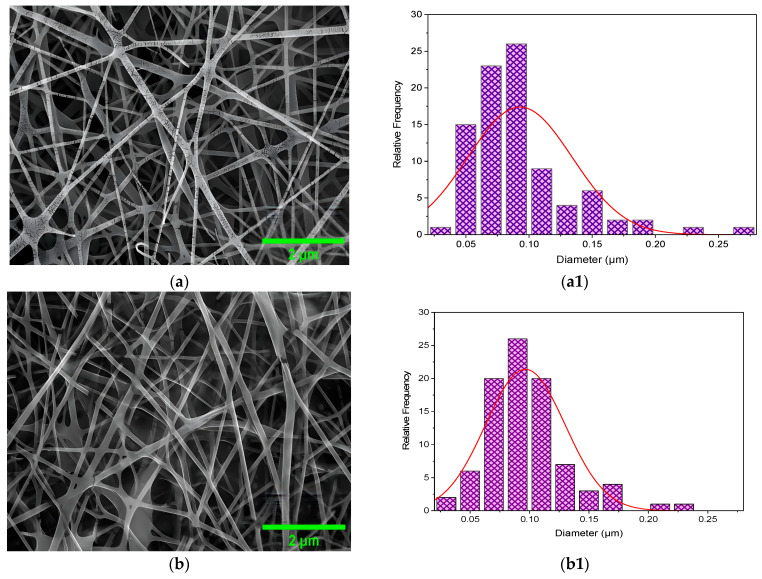

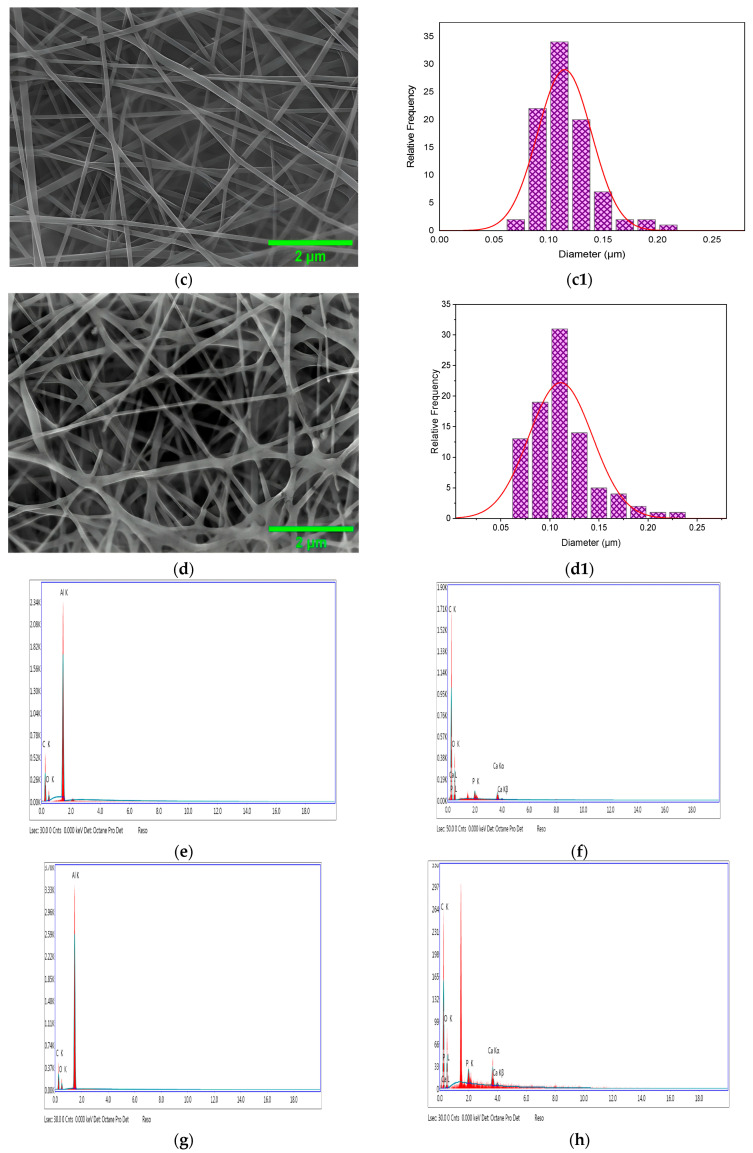
FE-SEM micrographs of the (**a**) CS/PVA, (**b**) CS/PVA/HA, (**c**) CS/PVA/Cur, and (**d**) CS/PVA/HA/Cur, (**a1**–**d1**) the fiber size distribution histogram, and (**e**–**h**) the EDS.

The microstructure and the size distribution of the electrospun nanofibers of CS/PVA, CS/PVA/HA, CS/PVA/Cur, and CS/PVA/HA/Cur mats produced at optimized conditions are shown in Figure 5a–d,a1–d1, respectively. The microstructure of all samples was marked by a uniform and smooth morphology with no surface presence of hydroxyapatite and curcumin aggregates, meaning that they are perfectly incorporated into the nanofibers. Pure CS/PVA nanofiber mats have an average diameter of ~90 nm, whereas CS/PVA/HA, CS/PVA/Cur, and CS/PVA/HA/Cur nanofiber mats have an average diameter of 96, 114, and 110 nm, respectively, see Figure 5a1–d1.

These results can be explained as follows: The addition of either curcumin or HA or both of them increases the viscosity of the polymer solution, which results in a higher viscoelastic force in the polymer jet that resists the stretching repulsive force of electric charges, resulting in a slight increase in the diameter of the fibers [23,24]. However, this incorporation of HA and curcumin did not alter the fiber diameter significantly. Nanofibers, which guarantee a moist environment to encourage wound healing (in several types of wounds) with scar inhibition and limit bacterial penetration, are present in all samples and offer suitable conditions for gas exchange [24,34,50]. Additionally, in all formulations, the surface is comparatively smooth and devoid of drug crystals, indicating that the drug has completely integrated into the polymer matrix through the electrospinning process.

The corresponding energy dispersive X-ray (EDS) analyses of the CS/PVA, CS/PVA/HA, CS/PVA/Cur, and CS/PVA/HA/Cur blends are shown in Figure 5e–h. The analyses show that the presence of carbon and oxygen atoms in the CS/PVA and CS/PVA/Cur blends, along with the presence of calcium and phosphorus atoms in the case of CS/PVA/HA and CS/PVA/HA/Cur blends, confirms the purity of the prepared mats. The appearance of the Al atom in the analysis refers to the materials of the background of the fibers (the materials of the collector).

**Figure 6:**
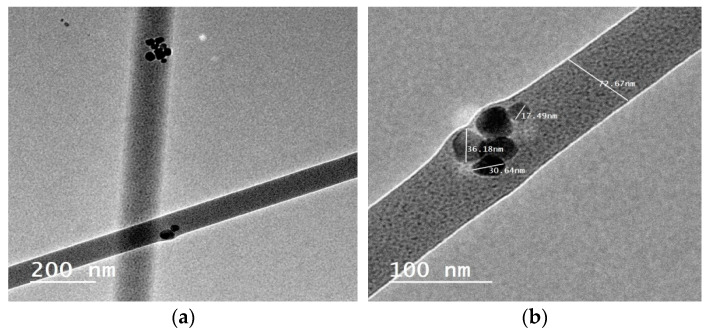
HR-TEM micrographs of the (**a**) CS/PVA/HA/Cur and (**b**) incorporation of nano-particles of HA within CS/PVA/HA/Cur nanofibers.

High-resolution transmission electron microscopy (HR-TEM) is also used to study the morphology of the CS/PVA/HA/Cur nanofiber mat. Selected HR-TEM micrographs are used to visualize the HA and Cur in the CS/PVA nanofiber mat. Figure 6a,b show that HA and curcumin are embedded within the CS/PVA nanofiber mat, with an average diameter of 80 nm. The semi-spherical HA nanoparticles within the nanofibers can be clearly seen, with sizes ranging from 10 to 50 nm, see Figure 6b.

#### 3.2.2. Composition of the As-Spun Mats

##### XRD Analysis

**Figure 7:**
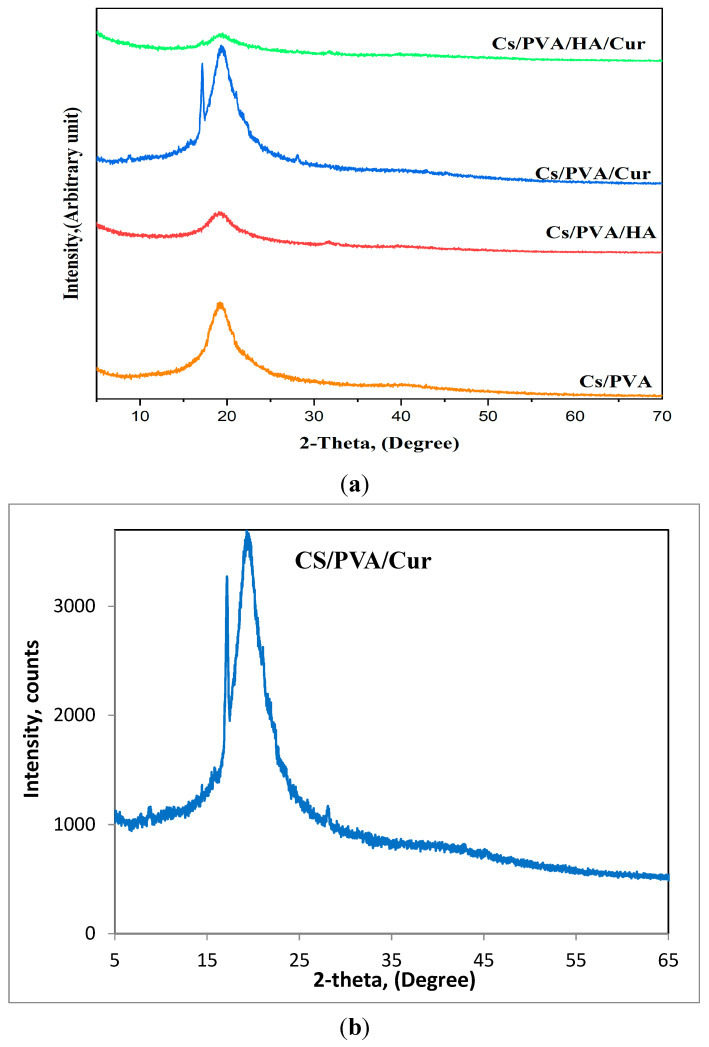
(**a**) XRD patterns of all fabricated mats, (**b**) XRD pattern of CS/PVA/Cur for further illustration.

The fabricated mats are tested for their crystallinity via XRD analysis. The CS/PVA diffraction pattern, as depicted in Figure 7a, exhibits the characteristic peaks of both CS and PVA. A broad peak is detected at around 20°, due to the peaks’ overlapping of PVA and chitosan, indicating the successful blending of PVA and chitosan.

Along with the distinctive peak of the CS/PVA blend at 20°, the XRD pattern of CS/PVA/HA reveals the existence of a broad diffraction peak between 31° and 32°, demonstrating the presence of the HA phase. Based on the polymer content of the mat nanofibers, the small amount of HA (5 wt%) may be the reason for this drop in HA crystallinity, as seen by the broader HA peak.

A similar behavior is also observed for the CS/PVA/Cur mat, where the XRD pattern shows some of the characteristic diffraction peaks of curcumin at about 8.7, 17.7, and 28°, but with very low intensity, which can be attributed to the incorporation of curcumin within semi-crystalline PVA, which may cause curcumin to lose its crystalline nature [25]. To enhance clarity, the XRD pattern of CS/PVA/Cur, see Figure 7b, is included for further illustration. The XRD pattern of the CS/PVA/HA/Cur mat contains only the main characteristic peak of HA and Cur, referring to the encapsulation of HA and Cur.

##### Fourier Transform Infrared Spectroscopy

**Figure 8:**
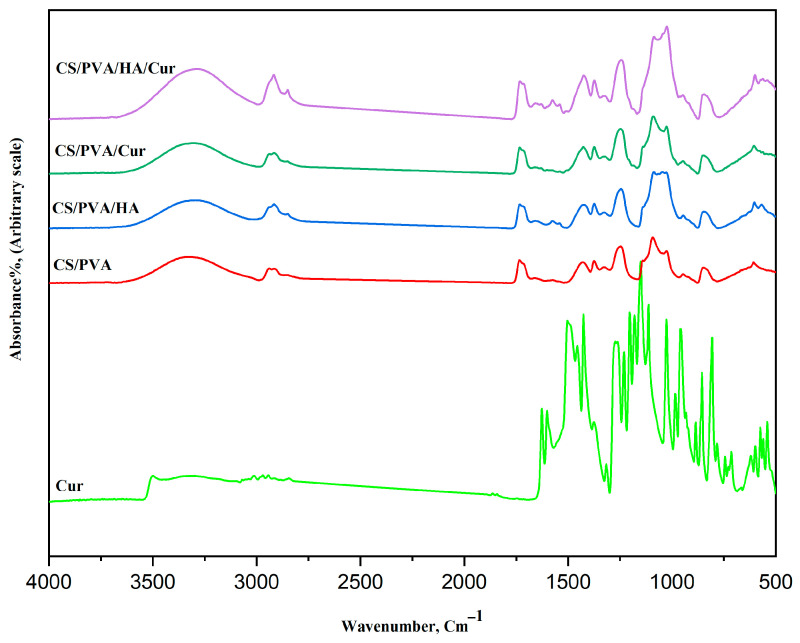
FT-IR spectra of curcumin and the different fabricated mats.

The FT-IR spectroscopy has been carried out to confirm if there is physical or chemical bonding occurring in the prepared mats, and the results of the FT-IR spectra for curcumin, CS/PVA, CS/PVA/HA, CS/PVA/Cur, and CS/PVA/Cur/HA, are presented in Figure 8. In the spectrum of curcumin, the absorption bands at 3301–3014 cm^−1^ are assigned to the hydroxyl group in curcumin. While the bands at 1601–1626 cm^−1^ refer to the stretching of carbonyl groups, which are the characteristic bands of curcumin. Also, the absorption bands between 1626 and 1375 cm^−1^ can be ascribed to the presence of aromatic and aliphatic C=C vibrational modes. The band at 1273 cm^−1^ indicates the aromatic C-O stretching vibration. The bands at 1151 and 1025 cm^−1^ are due to the presence of an ether group (C-O-C) [3,4,5].

The absorption bands at 855 and 807 cm^−1^ represent the C–H out-of-plane bending for 1,2,4-substituted aromatic rings.

All four mats show similar characteristic absorption bands of both PVA and chitosan. The bands of PVA and CS are in agreement with the literature data [25,34,35].

The broad band at 3500–3315 cm^−1^ is caused by O-H and N-H stretching vibrations, as indicative of the overlapping between CS and PVA. Bands at 2939–2915 cm^−1^ are ascribed to the C-H stretching vibrations of PVA. Bands at 946 and 847 cm^−1^ are observed, which are related to CH_2_ rocking and C-O stretching of PVA, while the band at 1092 cm^−1^ is due to the vibration of C-O bonds in the PVA. Two bands at 1374 and 1430 cm^−1^ are ascribed to CH-OH and CH_2_ symmetric bending mode vibrations of PVA. The band at 1733 cm^−1^ is related to stretching vibrations of the C=O groups of the remaining vinyl acetate group that is un-hydrolyzed in the PVA.

The characteristic absorption bands of CS at 1659 cm^−1^ (stretching of C=O amide I), 1574 cm^−1^ (amide II), and 1327 cm^−1^ (amide III) are present. The symmetric, and asymmetric -CH_2_- stretching modes of the pyranose ring are observed at 2915, 2850, 1430, 1327, and 1246 cm^−1^.

The bands at 1374 and about 1430 cm^−1^ are due to the -CH_3_ symmetrical deformation mode.

Moreover, the bands at the 1200–1000 cm^−1^ region are assigned to polysaccharide structures. Additionally, the band at about 1025 cm^−1^ is attributed to the C-N stretching vibration.

There is an obvious shift in the position of those characteristic absorption bands of pure PVA and chitosan, which indicates a physical bonding between them through hydrogen bonding.

The spectra of CS/PVA and CS/PVA/HA are similar concerning the characteristic functional chemical groups of CS/PVA and HA, i.e., almost all the characteristic bands of HA exist in the CS/PVA/HA spectrum, which is indicative of a successful loading of HA.

However, for the CS/PVA/Cur spectrum, some of the characteristic spectral bands of curcumin have disappeared, which indicates a significant physical overlap between the CS/PVA and curcumin spectra, i.e., successful encapsulation of curcumin within chitosan and polyvinyl alcohol.

### 3.3. Antimicrobial Activity

**Figure 9:**
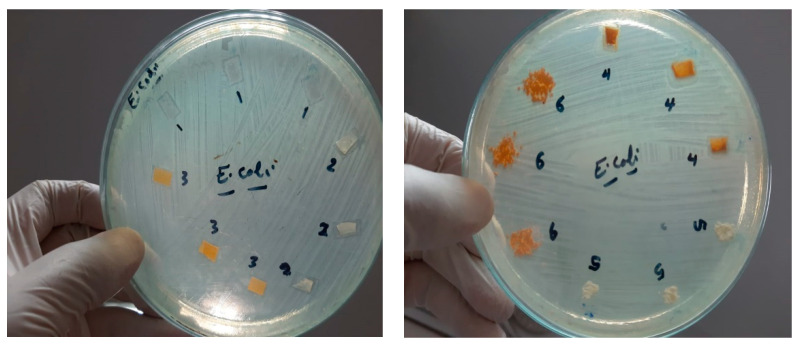

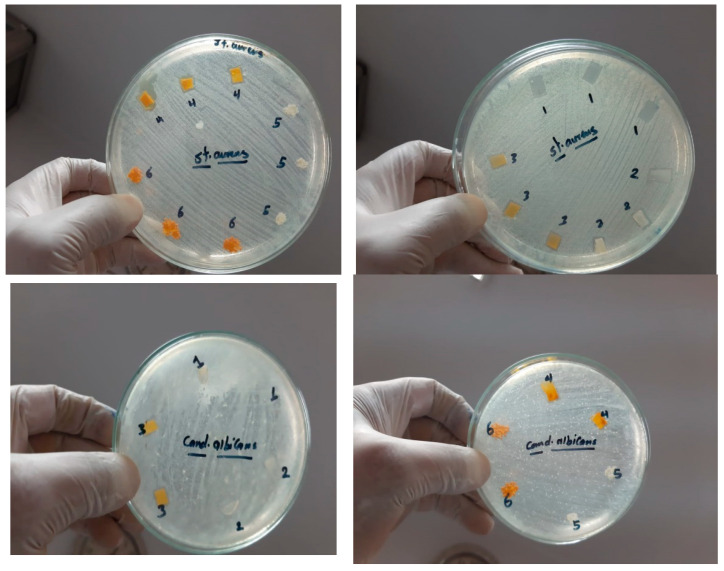
Inhibition zone of different mats against *E. coli*, *S. aureus*, and *C. albicans*, 1 = CS/PVA, 2 = CS/PVA/HA, 3 = CS/PVA/Cur, 4 = CS/PVA/HA/Cur, 5 = HA powder, and 6 = Cur powder.

As the antimicrobial activity of the mats plays an important role in wound dressing purposes, the fabricated mats were studied against *E. coli*, *S. aureus*, and *C. albicans*. The results of the antimicrobial activity of the nanofibers are shown in Figure 9, which shows the inhibition zones. Most of all the fabricated mats represent antimicrobial activity against the studied bacteria as indicative of the presence of an inhibition zone; however, in the case of CS/PVA/HA/Cur, no clear inhibition zone is created for E. coli. Due to this mat, cannot pass the bacterial cell membrane of E. coli and enter inside it, so no activity is recorded.

On the other hand, all the fabricated mats have no antifungal activity, as shown in Figure 9. Table 1 summarizes the diameter inhibition zone for all mats. The diameter of the inhibition zone for the CS/PVA/HA mat against *E. coli* and Staphylococcus aureus is 12.3 and 14 mm, respectively, whereas it is 11.6 and 14.3 mm, respectively, for the CS/PVA/Cur mat, which proves the improvement in the antibacterial effect of those mats compared to the CS/PVA mat. As shown in Figure 9 and Table 1, the diameter of the inhibition zone created by the CS/PVA mat is 11.6 and 13 mm in the case of *E. coli* and *S. aureus*, respectively. It is worth mentioning that the CS/PVA/HA/Cur mat shows less antibacterial effect against *S. aureus* as compared to other fabricated mats.

The antibacterial effect of chitosan works through its positive charge on its polymeric chain, which can electrostatically bind to the negative charge of bacterial cell walls to disrupt the cell membrane, leak DNA out of the matrix, bind with chitosan to inhibit DNA replication, and ultimately kill bacteria [1,36].

Curcumin has well-established antimicrobial activity. The mechanism by which chitosan exerts its antibacterial activity is via the inhibition of bacterial growth, damage to their cell walls, and prevention of transportation across their membranes [26].

On the other hand, HA can influence the bacteria through inducing Ca^2+^ ions stress, which subsequently disrupts the bacterial cell membrane. Moreover, the nano-nature of the components forming the nanofibers increases their bioavailability because the high surface area of the nanofiber enables direct interaction with the cell membrane and, hence, increases permeability.

The structural variations in the periplasmic space of different bacterial strains could account for the observed results. Gram-negative bacteria are characterized by a complex cell wall, which improves their resistance to chemical agents. Furthermore, Gram-negative bacteria can prevent the accumulation of antibacterial agents within the cell membrane through the presence of numerous efflux pumps [51,52].

The results show that those nanofibers could be suitable for use as a wound dressing with protective, healing, and antimicrobial effects. This shows that the wound healing patch is effective against both Gram-negative and Gram-positive bacteria. All the fabricated mats exhibit antibacterial activity against all studied bacteria, except CS/PVA/HA/Cur against *E. coli*. CS/PVA/Cur mats exhibit the strongest antibacterial activity against *S. aureus* and a comparable antibacterial activity to the CS/PVA mat against *E. coli*. The results of the antibacterial activity demonstrate that the fabricated nanofibrous mats are a promising material for wound dressing patches.

### 3.4. In Vitro Release Profile of CS/PVA/Cur and CS/PVA/HA/Cur Nanofiber Mats and Kinetic Study

**Figure 10:**
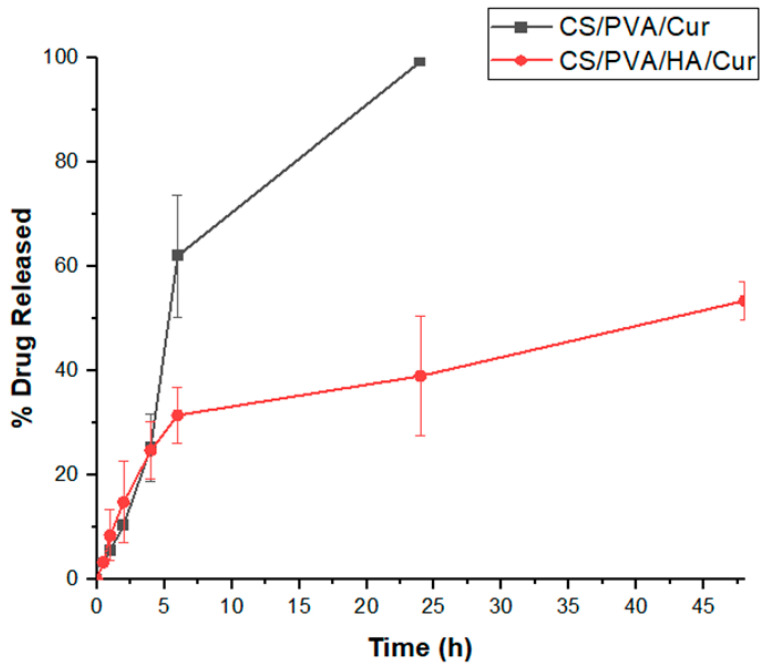
Cumulative release of curcumin from CS/PVA/Cur and CS/PVA/HA/Cur nanofiber mats.

In vitro drug release profiles of electrospun CS/PVA/Cur and CS/PVA/HA/Cur nanofiber mats were evaluated in PBS (pH = 7.4) with the addition of 0.5% *w*/*v* of Tween^®^ 80, to maintain sink conditions for curcumin release given its hydrophobic nature [53]. As shown in Figure 10, both formulations display a release profile characterized by an initial burst release, followed by a sustained release. Specifically, 62.06% ± 11.65 and 31.53% ± 5.39 of curcumin was released from CS/PVA/Cur and CS/PVA/HA/Cur, respectively, in the first 6 h, probably due to the drug molecules present on the fiber surfaces, which rapidly dissolved into release medium [47]. Instead, the fraction of curcumin entrapped within the nanofibers was released at a sustained rate [27], until it reached its maximum value after 24 h, for CS/PVA/Cur (99.37% ± 0.35), and 48 h of release, for CS/PVA/HA/Cur (53.49% ± 3.65), respectively. It is worth noting that nanofibers containing hydroxyapatite (HA) offer enhanced control over curcumin release, primarily due to the diffusion of Cur from the “double carrier”, as already reported in the literature [54]. In this process, Cur is first released from HA into the polymer matrix (CS/PVA) and subsequently diffuses into the release medium, resulting in a significantly slower and more sustained release profile. Palanisamy et al. [55] similarly observed that Cur loaded onto HA-nanoparticles demonstrates gradual release, attributed to its physical adsorption on the HA surface. This interaction is further explained by curcumin’s ability to chelate metal ions, which strengthens its adhesion to the polymer matrix through calcium ions in HA, further delaying its release from the nanofiber structure [28].

Moreover, to assess the release mechanism of curcumin, first-order, Higuchi, Korsmeyer–Peppas, and Peppas–Salhin models were used to fit the drug release data. Table 2 reported the mathematical model equations, their kinetic constants, and the adj-R^2^. In particular, k_1_ is the first-order rate constant, K_H_ is the dissolution constant, k is the release-rate constant, K_d_ is the Fickian diffusional contribution, and K_r_ is the matrix swelling or erosion contribution.

**Figure 11:**
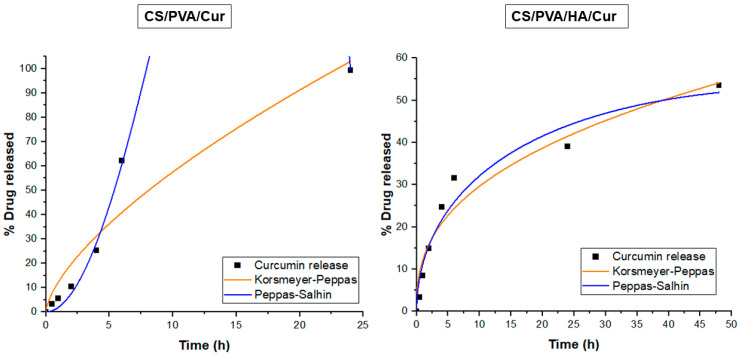
Release data of CS/PVA/Cur and CS/PVA/HA/Cur nanofiber mats fitting to Korsmeyer–Peppas and Peppas–Salhin models.

According to Adj-R^2^ values (Table 2), curcumin release from both CS/PVA/Cur and CS/PVA/HA/Cur nanofiber mats principally follows Korsmeyer–Peppas and Peppas–Salhin models (Figure 11). In the Korsmeyer–Peppas model, the n value < 0.45 indicates that the release of curcumin from CS/PVA/HA/Cur nanofibers was Fickian diffusion, while the n value > 0.45 for CS/PVA/Cur formulation suggests an anomalous curcumin transport, where in addition to the diffusion other mechanisms contribute to its release, such as the PVA matrix’s swelling and/or erosion [25]. The Peppas–Salhin model related to the release of curcumin from CS/PVA/HA/Cur nanofibers confirms the Fickian diffusion as a release mechanism since the K_r_ assumes a non-significant negative value. As concerns the curcumin release from CS/PVA/HA/Cur nanofiber mats, it is worth noting that the contribution of the matrix erosion/swelling is not significant since the $K_{r}$ value is close to zero.

Therefore, in these matrices, the diffusion mechanism of curcumin from both CS/PVA and CS/PVA/HA nanofibers prevails on the polymer swelling/erosion mechanism [51].

### 3.5. Evaluation of Cell Viability

**Figure 12:**
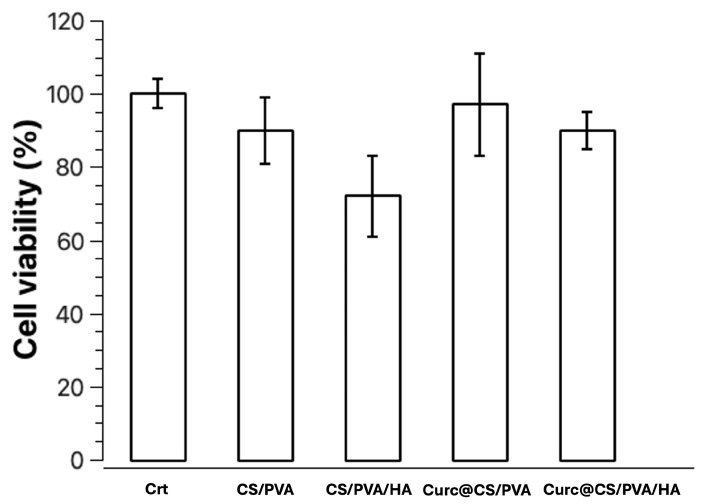
Cell viability of NIH-3T3 cells after 24 h in the presence of the samples CS/PVA, CS/PVA/HA, CS/PVA/Cur, and CS/PVA/HA/Cur. For each sample set the results are normalized on the viability at 24 h for the sample. Data represent the mean ± SD.

NIH-3T3 cells were used to evaluate the cell response to the presence of the CS/PVA, CS/PVA/HA, CS/PVA/Cur, and CS/PVA/HA/Cur composite. The cell viability on the different composite materials was tested using fibroblasts at 24 h, as reported in Figure 12. The results were normalized to the viability of the sample. Compared to cells, the CS/PVA sample has a decrease of only 10%. The sample with added HA in the CS/PVA composite does not lead to an increase in cell viability; in fact, we have a reduction of about 28%. The CS/PVA samples loaded with curcumin have a fibroblast viability that is unvaried compared to the control. So, the presence of HA and Cur is not toxic to fibroblasts, but cell viability increases slightly in the presence of curcumin.

## 4. Conclusions

In this study, electrospun nanofibers of CS and PVA blends loaded with nano-hydroxyapatite and/or curcumin are successfully fabricated by the electrospinning method for the first time. The fabricated mats are investigated by FE-SEM, TEM, and FT-IR as well as XRD. It has been proved that encapsulation of nano-hydroxyapatite and/or curcumin within the CS/PVA nanofibers enhances both cell viability and release of curcumin; moreover, the result of the in vitro antimicrobial test shows that most of mats have significant antimicrobial effects against *E. coli* and *S. aureus*; accordingly, these mats can be used instead of chemical antimicrobials, or along with them. Hence, promising novel scaffolds could be a potential candidate for wound dressing applications.

## Figures and Tables

**Table 1:** Inhibition Zone of different mats against *E. coli*, *S. aureus*, and *C. albicans*.

		Samples	No.	1			2			3			4			5			6		
Test Bacteria			Code	A	b	c	a	b	c	a	b	C	a	b	c	a	b	c	A	b	c
1	*E. coli*			11	13	11	11	12	14	11	12	12	-	-	-	-	-	-	-	-	-
2	*S. aureus*			13	12	14	15	14	13	15	15	13	12	11	13	-	-	-	-	-	-
3		*C. albicans*		-	-	-	-	-	-	-	-	-	-	-	-	-	-	-	-	-	-

**Table 2:** Correlation coefficient (R^2^) values according to First-order, Higuchi, Korsmeyer–Peppas, and Peppas–Salhin models and related kinetic constants.

Sample	Mathematical Model	Kinetic Constants	Adj-R^2^
CS/PVA/Cur nanofiber	First-order: $\frac{M_{t}}{M_{0}}=e^{-k_{1}t}$	$k_{1}=$ 0.85 ± 0.28	0.46
	Higuchi: $\frac{M_{t}}{M_{0}}=K_{H}\surd t$	$K_{H}=$ 18.99 ± 2.10	0.88
	Korsmeyer–Peppas: $\frac{M_{t}}{M_{0}}={kt}^{n}$	k= 2.2 ± 0.8 n= 1.8 ± 0.2	0.91
	Peppas–Salhin: $\frac{M_{t}}{M_{0}}={K_{d}t}^{m}+{k_{r}t}^{2m}$	$K_{d}=$ 2.1 ± 0.4 $K_{r}=$ 0.01 ± 0.02	0.99
CS/PVA/HA/Cur nanofiber	First-order: $\frac{M_{t}}{M_{0}}=e^{-k_{1}t}$	$k_{1}=$ 0.86 ± 0.14	0.68
	Higuchi: $\frac{M_{t}}{M_{0}}=K_{H}\surd t$	$K_{H}=$ 8.43 ± 0.61	0.91
	Korsmeyer–Peppas: $\frac{M_{t}}{M_{0}}={kt}^{n}$	k= 12.25 ± 1.99 n= 0.38 ± 0.05	0.94
	Peppas–Salhin: $\frac{M_{t}}{M_{0}}={K_{d}t}^{m}+{K_{r}t}^{2m}$	$K_{d}=$ 11.88 ± 2.49 $K_{r}=$ −0.66 ± 0.26	0.94

## Data Availability

The data presented in this study are contained within the article.
